# Urologic Issues During Pregnancy

**DOI:** 10.1100/tsw.2004.92

**Published:** 2004-06-07

**Authors:** Jeffrey P. Weiss

**Affiliations:** Clinical Adjunct Assistant Professor of Urology, New York Hospital-Cornell Medical Center, USA

**Keywords:** Pregnancy, physiology, hydronephrosis, calculi, infection, renal failure, transplantation

## Abstract

Pregnancy induces a variety of physiologic changes in the urinary tract. When such changes become accentuated the physiologic becomes the pathologic and symptoms arise, at times of significance enough to threaten the well being of mother and/or fetus. This article intends to describe the basis for urinary physiology and its pathologic counterparts during pregnancy. Such a background may then facilitate a rational management protocol for various urologic problems in the gravid state.

## URINARY TRACT SYMPTOMS DURING PREGNANCY

Frequency of voiding and stress incontinence are the most common urinary symptoms experienced by the gravid patient. Additional symptoms include urinary urgency, urge incontinence, incomplete emptying, and slow stream (1,2). Hematuria is a physiologic change of pregnancy owing to microanatomic alterations in venous fragility of the collecting tubules (3). Flank pain may be due to accompanying processes such as calculi, hydronephrosis, pyelonephritis or spontaneous renal rupture, a phenomenon peculiar to pregnancy.

### Pregnancy-Induced Changes in Renal Physiology

Hydroureteronephrosis is the most significant renal functional alteration of pregnancy, accounted for by both hormonal and mechanical factors. Increases in circulating estrogenic, progestational and prostaglandin-like agents are known to cause ureterectasis in the absence of obstruction during pregnancy (4,5). Stasis caused by dextrorotation of the expanding uterus is the principal mechanical factor leading to hydroureteronephrosis of pregnancy and explains its tendency to occur on the right.

Glomerular filtration rate (GFR) and renal plasma flow (RPF) are ordinarily increased during pregnancy (6). Both of these changes are attributable to increases in cardiac output, decreases in renal vascular resistance and increases in serum levels of progesterone, aldosterone, deoxycorticosterone, placental lactogen and chorionic gonadotropin (7). Increased urinary excretion of glucose, amino acids, protein and vitamins are consequent to increases in RPF and GFR (8, 9).

Renal volume is seen to increase during pregnancy up to 30% normal size, a result of increased RPF and glomerular surface area under the influence of prolactin and its growth hormone-type of effect (10). In addition, we see hypercalciuria as a concomitant of pregnancy due to increased GFR, calcium filtration and intestinal calcium absorption related to high levels of plasma calcitriol (11, 12). However, an increase in inhibitors of stone-formation such as citrate, magnesium and glycosaminoglycans yields the result that pregnancy is unassociated with a net change in rates of stone formation (13, 14).

While increases in GFR of pregnancy would ordinarily cause the loss of 5,000 to 10,000 mEq sodium daily (15), there are mitigating factors resulting in maintenance of sodium homeostasis among which are tubular reabsorption (16), and increases in mineralocorticoid-like compounds such as aldosterone (17), deoxycorticosterone (18), estrogens (19), ACTH, prolactin, cortisol, growth hormone and placental lactogen (20). The influence of the renin-angiotensin axis upon sodium homeostasis is mediated by aldosterone metabolism and volume status (21). Physical factors affecting sodium regulation during pregnancy are increases in ureteral pressure and uterine blood supply. Thus, the net effect of the multiple aforementioned factors upon sodium metabolism is a slight sodium retention in pregnant subjects.

## HYDRONEPHROSIS OF PREGNANCY

Gestational upper urinary tract dilatation results from evolution of a physiologic process, namely, ureteral compression by the expanding uterus (22). Because hydroureteronephrosis of pregnancy occurs as an outcome of dextrorotation of the uterus at mid-term, it usually happens on the right (23,24). Ureterectasis is also thought to have a non-obstructive component, related to hormonal changes induced by a functioning placenta (25,26).

Painful hydronephrosis of pregnancy is conventionally treated by having the patient remain situated to the left in order to relieve ureteral pressure induced by the dextrorotated uterus (fig 1a, 1b). When this measure fails, ureteral stenting or establishment of percutaneous drainage are effective in pain relief and preventing evolution of hydronephrosis to spontaneous renal rupture (28-32). Alternative resolutions to the problem of symptomatic gestational hydronephrosis include induction of labor and delivery (33,34), and epidural block (35).

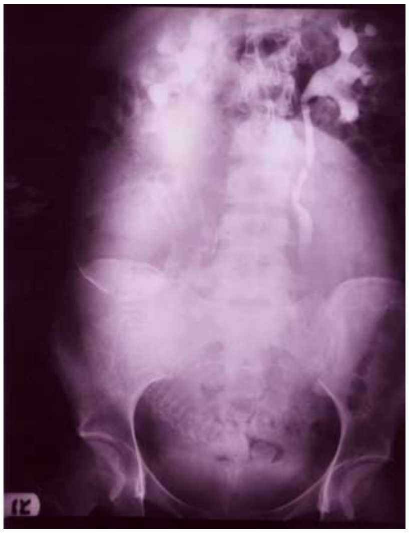

**FIGURE 1a**. Urogram, third trimester patient complaining of right flank pain, 15 minute film demonstrates bilateral hydronephrosis.

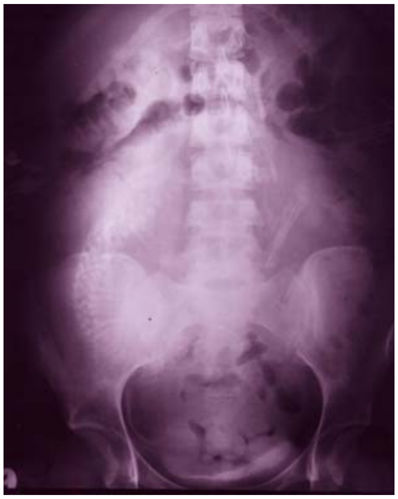

**FIGURE 1b**. Same patient, 2.5 hour film demonstrates near-total drainage of all contrast. Diagnosis: Symptomatic right hydronephrosis of pregnancy, moderate without evidence true obstruction. Pain resolved with bedrest in the left contralateral position.

Spontaneous renal rupture (SRR) is a potentially fatal complication of unchecked gestational obstructive uropathy, thought to be occasioned by increased hydrostatic pressure leading to extravasation from overstressed calyceal-renal capsular junctions (36). A recent review of non-traumatic rupture of the gravid urinary tract reveals that it is uncommon, with 25 reported cases to 1995 (27). The majority of cases occurred in diseased kidneys, typically harboring tumors. About half the renal units ruptured through the collecting system and half through parenchyma. Also at risk are renal units rendered less than normally compliant due to prior infection, surgery or trauma. While SRR occurs between 18 weeks of pregnancy and 1 day post-parturition (37), this phenomenon is accompanied by symptoms of loin pain, hematuria, flank mass and hypotension. Diagnosis is made through the use of ultrasound, excretory urography and retrograde studies. Arteriography may be both diagnostic (in identification of the vessel or vessels responsible for hypotension) and therapeutic in such instances (angioembolization), though relatively contraindicated in the first trimester because of high radiation dosages necessary in most cases (38).

## DECISION-MAKING FOR GESTATIONAL URINARY TRACT IMAGING

Significant pelvic radiation dosages (5-15 cGy) during the first trimester increase the risk of teratogenicity from 1 to 3 percent (39). Putting this into perspective, a standard urogram renders 1.5 cGy to the fetus; however, prudence dictates the standard that limited exposure to 2 or 3 Îshotsâ should be taken during a gestational urogram. These images include the plain film, a thirty minute exposure and 2-3 hour exposure if the diagnosis of obstruction remains in doubt. Since each plain abdominal film yields 0.2 cGy to the fetus, the 2 or 3 shot urogram is considered safe even during the first trimester (40-42). On the other hand, data exists to cause concern with even low doses of roentgen ray exposure to the fetus. Specifically, an average of 1 rad of fetal exposure has been correlated with a net 2.4-fold increase in the incidence of all childhood malignancies (43). However, it is not clear during which trimesters radiation exposure occurred. While ultrasound is safe under all circumstances of pregnancy, its utilization in diagnosing obstruction is of limited value owing to its suboptimal view of the ureter and presence of hydroureteronephrosis as a physiologic concomitant of pregnancy. Thus, because urography is found to have diagnostic value greatly in excess of sonography during pregnancy (44), it is recommended in the following situations: [a] persistent fever [b] massive or increasing hydronephrosis as seen during serial urosonography [c] pain and or emesis refractory to conservative therapy. The theoretical risk of development of childhood cancer (not necessarily teratogenicity) should at all times be kept in mind when subjecting the fetus to roentgen ray exposure (45,46). In particular, there is no evidence that fetal radiation exposure below 5 rad causes congenital malformation, spontaneous abortion or growth retardation (47). However, the relative risk of childhood leukemia occasioned by fetal exposure of 1-2 rad is increased from 1/3000 (general population) to 1/2000 (48). Put into perspective, the risk of leukemia for a sibling of a leukemic child is 1/700 (49).

In view of these data, it is useful to discuss radiation dosages received by the uterus owing to specific imaging studies. For example, cerebral angiography causes less than 10 mrad exposure to the uterus, a negligible dosage, due to the maximum distance of the collimated beam from the brain to the pelvis (50). In contrast, double vessel coronary angioplasty provides 90 mrad uterine exposure (51,52) and barium enema causes 2-4 rad fetal exposure due to proximity of the study to the uterus (53). Other roentgen ray studies may be ordered during pregnancy. Computed tomography causes maximum radiation exposure at the skin level with a progressive decrease toward the body interior. Correspondingly, radiation dosage to the fetus diminishes with enlargement of the pregnancy and its investing tissues (54). Specifically, a 10 slice abdominal study causes 2.6 rad fetal exposure at weeks 0-14 whereas the same study provides only 1.7 rad to the conceptus at weeks 35-42 (55-57). Likewise, administration of radiopharmaceuticals may be safely done during pregnancy. Nuclear medicine studies of the brain, biliary system, skeleton, lungs, kidneys, abscesses and heart may all be accomplished with fetal exposures varying from 40 to 1100 mrad (58). In general, because radioactive iodine readily crosses the blood-placental barrier, isotopic iodine administration should be avoided during pregnancy (59). Magnetic imaging is felt to be safe during pregnancy in that studies of static, gradient and radiofrequency magnetic fields at strengths lower than 2 tesla have thus far failed to demonstrate mutagenic or other deleterious effects upon the fetus (60-63).

In summary,it is useful to keep in mind the American College of Obstetricians and Gynecologists' guidelines for diagnostic imaging during pregnancy (64):

- Counseling that x-ray exposure under 5 rad has not been associated with increased in fetal anomaly or spontaneous abortion.
- Maternal health should not be compromised by irrational fears of the dangers of ionizing radiation to the fetus. However, alternative imaging procedures such as ultrasonography and magnetic resonance imaging should be employed instead of x-rays where applicable.
- While ultrasonography and magnetic resonance imaging are unassociated with known adverse fetal effects, magnetic resonance imaging to date is not recommended for use in the first trimester.
- Radiologic consultation is advisable if it is deemed necessary to estimate fetal dose when roentgenologic procedures are performed during pregnancy.
- Therapeutic radioactive iodine isotopes are contraindicated during pregnancy.

## URINARY CALCULOUS DISEASE DURING PREGNANCY

Because factors which tend to increase stone formation are balanced by inhibitory influences during pregnancy, pregnant patients are no more likely to manufacture urinary calculi than their nonpregnant counterparts. Specifically, decreased ureteral peristalsis, hydronephrosis, infection and calcium supersaturation all enhance the stone-forming propensity in pregnancy, whereas augmented excretion of stone inhibitors such as citrate, magnesium and glycosaminoglycans tend to neutralize the former stone-enhancing factors (65,66).

As in all other patients, calculous disease of pregnancy is accompanied by symptoms of flank or abdominal pain, nausea, vomiting and lower urinary urgency. Because most calculi during pregnancy are located in the ureter, sonography will usually be insufficient to make the diagnosis. Thus, in accordance with the above discussion, roentgenographic imaging studies are necessary to diagnose many symptomatic gestational urinary tract calculi.

The primary treatment of stones in pregnant subjects is expectant, as most will pass spontaneously (67-71). Refractory cases will be treated with ureteral stenting with monthly stent changes due to the proclivity for calcific encrustation during pregnancy (72). Ureteroscopy (with or without laser lithotripsy) is facilitated by the compliant ureter of pregnancy and is helpful in resolving the problem of obstruction and pain without the need for morbid long-term stenting in these patients (73-76). My personal preference for ureteroscopy done without x-ray exposure is to cystoscopically pass a guidewire into the distal ureter which is usually straight even during the third trimester. Over this wire a 7 french steerable ureteroscope may be advanced under direct vision into the kidney at which time the wire may be placed confidently into the kidney. The calculus may then be basketed or destroyed with laser or electrohydraulic lithotripsy. The stent may be advanced through the cystoscope until a single pigtail remains in the bladder. If doubt exists a single (0.2 rad) plain film can confirm stent position. While stenting has been reported to be effective in the majority of cases (77), a large (6 or 7 french) stent should be used, as one series demonstrated relief of obstruction in the gravid ureter in less than 50% of cases (78).

Percutaneous nephrostomy and open lithotomy in selected circumstances continue to be legitimate options for treatment of gestational urinary calculi (79-81). On the other hand, the status of extracorporeal shock wave lithotripsy is unclear: despite the fact that female fertility is thought to be unchanged by ESWL (82), the lack of data pertaining to safety of shock waves upon the developing fetus renders pregnancy a relative contraindication to ESWL.

## URINARY RECONSTRUCTION AND PREGNANCY

Pregnancy is possible in patients having undergone prior urinary tract reconstruction, whether for neurogenic bladder, tumor or voiding dysfunction. Fenn et al (83) described 19 pregnancies in 18 women aged 21 to 36 years having undergone clam enterocystoplasty for intractable detrusor instability. Pajor et al (84) advocate lower urinary reconstruction with an ileocecal as opposed to ileal bowel segment in order to avoid uterine-induced mesenteric compromise during the course of pregnancy. Kennedy et al (*85*) reported upon successful pregnancies in 4 women with exstrophy having had flap vaginoplasty and creation of subsequent continent right colonic urinary reservoir with an orthotopic perineal stoma (Indiana pouch). The authors performed cesarian section and close monitoring for maternal or fetal distress in all cases. Creagh et al (86) reported 34 pregnancies in 27 women with reconstructed lower urinary tracts who underwent either vaginal or cesarian delivery, indicated by specific obstetrical considerations. The majority of their patients (28/34) in fact underwent successful vaginal delivery. Thus, patients having undergone lower urinary reconstruction may safely deliver either vaginally or via C-section; attendance of the urologist is essential in all cases.

## URINARY TRACT INFECTION IN PREGNANCY

Twenty to forty percent of pregnant women with asymptomatic bacteruria during the first trimester will become afflicted with pyelonephritis in the third trimester (87,88). In addition, eradication of bacteruria diminishes the incidence of pyelonephritis and its attendant associations with prematurity, growth retardation and low birth weight (89,90). Thus, it is considered axiomatic that asymptomatic bacteruria should be screened for, treated and then prophylaxed (91). As is the case for nonpregnant patients, a clean catch urine culture yielding greater than 100,000 colony forming units per cc is considered significant for purposes of treatment and antibiotic prophylaxis for the remainder of the duration of pregnancy (92). Screening for and treatment of asymptomatic bacteruria to prevent third trimester pyelonephritis has been demonstrated to be cost-effective with either culture or dipstick strategies in comparison to a no-screening policy (93). Urethritis due to Chlamydia trachomatis occurs in 50% of women with dysuria, pyuria and urinary frequency (94). In addition, chlamydial cervicitis was found in 21% of 11,544 women at their first prenatal visit, and untreated was associated with premature rupture of membranes as well as low birth weight and decreased survival (95). Neonatal complications of chlamydial infection included nasopharyngitis, pneumonitis and conjunctivitis. Thus, chlamydia is not 'normal flora' and should be treated when discovered during pregnancy with erythromycin 500 mg four times daily for seven to ten days (96). Screening of the lower genital tract in pre-termination patients has been advocated to reduce the 63% incidence of pelvic inflammatory disease in patients harboring both chlamydia and anaerobic vaginosis (97).

The bacteriology of urinary infection in pregnancy involves gram negative rods, notably, E. coli, Klebsiella/Enterobacter, Proteus, as well as gram positive cocci and enterococci (98). While pregnancy per se does not statistically affect susceptibility to urinary infection, diabetics are colonized more frequently with group B streptococcus (although the latter agent does not account for increased infection rate in the diabetic pregnant population) (99,100). An interesting aspect of pregnancy is a relative status of immunosuppression, presumably due to the presence of the "foreign" genome of the fetus. Petersson et al (101) reported significant decline in the immune response in pregnant subjects to acute pyelonephritis. That is, maternal serum and urine interleukin-6 and specific antibody responses to E. coli were diminished in comparison to the situation in their non-pregnant counterparts at diagnosis.

Treatment of bacteruria (whether symptomatic or not) of pregnancy entails initial eradication of the offending microorganism followed by establishment of a program of uroprophylaxis. Initial treatment ranges from single dose to three day to ten day courses of antibiotics known to be free of teratogenic side effects (102-105). A randomized, controlled study of patients with pyelonephritis of pregnancy demonstrated the adequacy of outpatient treatment using a 10 day course of oral cephalexin (106). Subsequent prophylaxis may take the form of the same uropharmaceutical administered as a single nightly dose throughout pregnancy or merely as a postcoital dose (107). Urinary bacteriologic studies should then be maintained routinely, for example, monthly during gestation. Positive cultures despite prophylaxis are then responded to in the same fashion as the initial positive culture. Antibiotics thought safe during pregnancy (barring allergy) are: penicillins, cephalosporins and erythromycin. Others may be safely used with certain caveats: nitrofurantoin (watch for hemolysis in breastfed infants with G-6-PD deficiency), aminoglycosides (monitor levels) and sulfonamides (cause hemolysis/kernicterus after 28 weeks if G-6-PD deficiency exists). All other antibiotics are either contraindicated or carry strong relative contraindications during pregnancy (108). Table 1 provides a comprehensive description of antibiotic use during pregnancy.

## LOWER URINARY DYSFUNCTION IN PREGNANCY

Urinary stress incontinence and frequency constitute the major lower tract symptoms attributable to the gravid state; in contrast, retention during pregnancy occurs uncommonly (109, 110). Because retention is related to bladder neck obstruction caused by uterine retroversion and posterior movement of the cervix (especially in the first trimester), treatment entails manual repositioning of the fundus or placement of a pessary (111,112).

Stress incontinence of pregnancy occurs as a result of myogenic and/or neurogenic damage to the urethral sphincter especially following dystocia or forceps delivery (113). This Îdirect traumaâ theory is complemented by an alternate mechanism, namely, intrinsic change in urethral smooth muscle function induced by pregnancy itself, rather than birth trauma. Such a concept is supported by data revealing that patients with stress incontinence exhibit progressive diminution in urethral closure pressure as pregnancy progresses as opposed to their pregnant but continent counterparts whose urethral length and closure pressures increase toward term (114,115). A hormonal etiology for pregnancy-induced stress incontinence has also been proposed, specifically, urethral collagen depolymerization and softening mediated by the corpus luteum hormone Relaxin (116). Recent experimental data supports the concept that pregnancy induces alteration of smooth muscle compliance and functional responses of the bladder neck and urethra to various forms of autonomic stimulation and relaxation occasioned by the profound hormonal changes which are part and parcel of the gravid endocrine milieu (117).

**TABLE 1**
**Antibiotic use in pregnancy**

| Antibiotic | Safety margin (barring allergy) |
|---|---|
| penicillins | safe |
| cephalosporins | safe |
| erythromycin | safe |
| nitrofurantoin | safe (hemolytic anemia in breastfed infants with G-6-PD deficiency) |
| aminoglycosides | safe: monitor serum levels/renal function |
| sulfonamides | safe until 28 weeks (thereafter risk hemolysis/kernicterus if G-6-PD deficiency) |
| trimethoprim | contraindicated in first trimester (teratogenic, fetal folate antagonist) |
| tetracyclines | contraindicated (fetal limb/dental dysgenesis) |
| chloramphenicol | contraindicated near term (fetal bone marrow depression, "gray syndrome") |
| isoniazid | causes congenital defects: infant encephalopathy |
| metronidazole | use with caution second/third trimesters only (?mutagenic) |
| amox/clavulanate | formal studies lacking |
| quinolones | contraindicated due to effects on fetal bone and cartilage |
| ketoconazole | contraindicated (teratogenic in rats; inhibits steroid synthesis) |

A related issue is pregnancy in patients having been previously treated for stress incontinence, whether due to urethral hypermobility or intrinsic sphincter deficiency. While those having undergone retropubic suspension or sling procedures may deliver without jeopardizing the repair via cesarian section (118,119), vaginal delivery may ensue without ill effect upon prior implantation of either periurethral collagen or the artificial urinary sphincter (120,121).

## PREGNANCY AND RENAL FAILURE

Acute and chronic renal failure are caused and exacerbated, respectively, by pregnancy. Related issues deal with the effects of pregnancy upon the patient with established renal failure treated with either dialysis or transplantation.

Acute renal failure of pregnancy is subcategorized etiologically into three groups: prerenal, renal and postrenal. Hypovolemia due to hyperemesis gravidarum and uterine hemorrhage are the principle underlying causes of prerenal azotemia of pregnancy (122). Hemorrhage in turn is caused by abortion, placenta previa and abruption. Failure to replenish appropriate volume components causes prerenal azotemia to evolve into acute tubular necrosis (ATN), the most frequent etiology of gestational ARF (the other significant etiology being renal cortical necrosis [RCN]). RCN results from disseminated intravascular coagulation (DIC, in the setting of amniotic fluid embolism, intrauterine fetal demise or abruption), transfusion reactions and sepsis (chorioamnionitis, septic abortion and pyelonephritis) (123-125). RCN and ATN differ in that the former is manifested by anuria and irreversible renal impairment followed by renal cortical calcification in the healing phase, whereas the latter infrequently presents with anuria and tends to resolve to satisfactory renal function (126).

Pregnancy may or may not adversely affect kidney function in patients with established chronic renal failure (CRF). For example, 35% of patients with serum creatinines greater than 1.6 mg/dl in one series developed rapid renal deterioration (127). Likewise, reflux nephropathy tends to be aggravated by pregnancy: eight of 20 patients with this condition developed endstage renal disease (ESRD) within two years of delivery or abortion (128). Jungers et al concluded that while pregnancy may proceed uneventfully in patients with established reflux nephropathy but normal blood pressure and serum creatinine, there is compromise in both fetal prognosis and maternal renal function if pregnancy progresses in the presence of azotemia (129). Mansfield et al found that patients having undergone vesicoureteral reimplantation as children were at higher than normal risk for intragravid urinary infection, but suffered no increase in the incidence of spontaneous abortion (130). Belman (131) recommends warning women with a history of childhood urinary infection that they are both at risk for bacteruria of pregnancy and require antibiotic prophylaxis regardless of the state of their reflux during pregnancy. On the other hand, patients with autosomal dominant polycystic kidney disease may be counseled that their renal function is unlikely to be altered by pregnancy (132).

Despite the potentially deleterious effects of pregnancy upon renal function in patients with established CRF, pregnancy is indeed a possibility in patients with ESRD on dialysis. While offspring of such patients have no increased likelihood of congenital anomalies, they tend toward low birth weights. Mothers with ESRD tend to experience worsening of both hypertension and residual renal function (133,134).

Whereas renal transplantation increases the chance for pregnancy from 1/200 (dialysis patient) to 1/50 (135), transplanted kidneys tend not (85% of cases) to suffer ill effects from the processes of gestation, labor and delivery (136,137). It is recommended that renal transplant patients seeking childbearing become pregnant two to five years following transplantation since pregnancy more than five years after transplantation resulted in a 75% incidence of serious renal injury, while the pregnancy itself fared better more than two years post-transplantation (138,139).

## URINARY TRACT TUMORS DISCOVERED DURING PREGNANCY

Despite pregnancy being an immunologically impaired state, the incidence of malignancy is similar to that in the general population (140). A wide variety of urologic tumors has been reported to occur during pregnancy (141). Specifically, renal cell carcinoma is the commonest renal neoplasm of pregnancy; since the latter is a condition of the young, angiomyolipoma occurs next in frequency (142). For the same reason, Wilms tumor is known in pregnancy (143). Bladder cancer during pregnancy may take the form of adenocarcinoma (144), transitional cell carcinoma (145,156) or squamous cell lesions (147). As electrical current may induce neighboring uterine contractions, obstetrical treatment to diminish uterine smooth muscle reactivity may be helpful in reducing the possibility of premature labor from electroresection and cautery. Similarly, laser phototherapy is useful to treat bladder tumors of pregnancy while eschewing prematurity.

Adrenal tumors such as pseudocyst (148) and pheochromocytoma (149,150) are extant during pregnancy. Key issues in pheochromocytoma of pregnancy include diagnostic conundra (symptoms and signs resemble those of preeclampsia leading to more than 50% mortality when undiagnosed); choice of alpha blockade (prazosin, to avoid teratogenicity of phenoxybenzamine); means of imaging (magnetic resonance imaging is especially useful for localizing pheochromocytoma and is free of ionizing radiation); timing of surgical resection (expeditiously); and route of delivery (vaginal preferred) (151-156).

## TIMING OF ANESTHESIA DURING PREGNANCY

General anesthesia in and of itself does not entail a risk of adversity to pregnancy (157). This holds especially true when the (nonobstetric) procedure is complication-free. A landmark study from Scandinavia evaluated 5405 incidental surgical procedures performed during all 3 trimesters of pregnancy. While the incidence of low birthweight and prematurity was greater in these patients as compared with a large cohort of pregnancies, there was no tendency toward congenital malformation in the operated group. It was concluded that there is an increased risk of prematurity following intragravid surgical procedures requiring general anesthesia which may be attributed to the underlying condition rather than the procedure or anesthetic itself (158,159).

Other considerations involving anesthesia during pregnancy include timing of semiurgent procedures which may not be postponed until parturition. There is evidence that nonobstetric surgical procedures are most safely performed during the second trimester owing to the increased risk of spontaneous abortion during the first trimester and induction of premature labor when procedures take place near term (160).

A related issue is the diminished requirement for both local and general anesthetics during pregnancy (161). Thus, dosages of inhalational anesthetics such as halothane and isoflurane should be reduced in pregnancy to compensate for the sedative effects of progesterone (162-164). Likewise, the requirement for local anesthetics is decreased during the first trimester due to increased cell membrane receptor sensitivity to these agents, again a progestational-mediated phenomenon (165,166).

## CONCLUSION

Urologic problems during pregnancy are often undertreated due to unfounded fears of causing fetal harm. An understanding of pathophysiologic changes in the urinary tract as well as appropriate use of antimicrobials, anesthetics, imaging studies and invasive procedures will lead to resolution of most such problems while providing a margin of safety for both mother and child.
